# Comparison of acute pneumonia caused by SARS-COV-2 and other respiratory viruses in children: a retrospective multi-center cohort study during COVID-19 outbreak

**DOI:** 10.1186/s40779-021-00306-7

**Published:** 2021-02-16

**Authors:** Guang-Li Ren, Xian-Feng Wang, Jun Xu, Jun Li, Qiong Meng, Guo-Qiang Xie, Bo Huang, Wei-Chun Zhu, Jing Lin, Cheng-He Tang, Sheng Ye, Zhuo Li, Jie Zhu, Zhen Tang, Ming-Xin Ma, Cong Xie, Ying-Wen Wu, Chen-Xi Liu, Fang Yang, Yu-Zong Zhou, Ying Zheng, Shu-Ling Lan, Jian-Feng Chen, Feng Ye, Yu He, Ben-Qing Wu, Long Chen, Si-Mao Fu, Cheng-Zhong Zheng, Yuan Shi

**Affiliations:** 1 Department of Pediatrics, General Hospital of Southern Theater Command of PLA, 111 Liuhua Road, Yuexiu District, Guangzhou, 510010 Guangdong China; 2 Department of Pediatrics, the Third People’s Hospital of Shenzhen, Shenzhen, 518100 Guangdong China; 3 Pediatric Intensive Care Unit, Wuhan Children’s Hospital, Tongji Medical College, Huazhong University of Science & Technology, Wuhan, 430010 China; 4 Pediatric Intensive Care Unit, Maternal and Child Health Hospital of Huangshi, Huangshi, 435000 Hubei China; 5 Department of Pediatrics, the Second People’s Hospital of Guangdong Province, Guangzhou, 510317 China; 6 Department of Pediatrics, the Third Affiliated Hospital of Zunyi Medical University (the First People’s Hospital of Zunyi), Guizhou, 563000 China; 7 Department of Pediatrics, the Eighth People’s Hospital of Guangzhou, Guangzhou, 510440 China; 8 Department of Pediatrics, the First Affiliated Hospital of Xinxiang Medical University, Xinxiang, 453100 Henan China; 9 Pediatric Intensive Care Unit, the Children’s Hospital Zhejiang University School of Medicine, Hangzhou, 310000 China; 10 Department of Emergency / Critical Medicine, Children’s Hospital of Nanjing Medical University, Nanjing, 210008 China; 11 Department of Pediatrics, the Second Affiliated Hospital of Guangzhou Medical University, Guangzhou, 510260 China; 12 Department of Medical information date room, General Hospital of Southern Theater Command of PLA, Guangzhou, 510010 China; 13 Department of Pediatrics, the First Affiliated Hospital of Jinan University, Guangzhou, 510632 China; 14 Department of Pediatrics, Maternal and Child Health Hospital of Yangjiang, Yangjiang, 529500 Guangdong China; 15 Department of Pediatrics, Shenzhen Hospital Affiliated to the University of Chinese Academy of Sciences, Shenzhen, 518107 Guangdong China; 16 Department of Pediatrics, Nanfang Hospital of Southern Medical University, Guangzhou, 510515 China; 17 Department of Pediatrics, Zhujiang Hospital of Southern Medical University, Guangzhou, 510280 China; 18 Department of Pediatrics, Military Hospital of 74 Group of PLA, Guangzhou, 510318 China; 19 Department of Neonatology, Children’s Hospital of Chongqing Medical University/Ministry of Education Key Laboratory of Child/Development and Disorders/National Clinical Research Center for Child Health and Disorders/Chongqing Key Laboratory of Pediatrics, Chongqing, 400014 China; 20 Department of Pediatrics, Zhongshan Boai Hospital, Zhongshan, 528403 Guangdong China; 21 Department of Pediatrics, Strategic Support Force Medical Center of PLA, Beijing, 100101 China

**Keywords:** Children, Severe acute respiratory syndrome, COVID-19, Viral pneumonia

## Abstract

**Background:**

Until January 18, 2021, coronavirus disease-2019 (COVID-19) has infected more than 93 million individuals and has caused a certain degree of panic. Viral pneumonia caused by common viruses such as respiratory syncytial virus, rhinovirus, human metapneumovirus, human bocavirus, and parainfluenza viruses have been more common in children. However, the incidence of COVID-19 in children was significantly lower than that in adults. The purpose of this study was to describe the clinical manifestations, treatment and outcomes of COVID-19 in children compared with those of other sources of viral pneumonia diagnosed during the COVID-19 outbreak.

**Methods:**

Children with COVID-19 and viral pneumonia admitted to 20 hospitals were enrolled in this retrospective multi-center cohort study. A total of 64 children with COVID-19 were defined as the COVID-19 cohort, of which 40 children who developed pneumonia were defined as the COVID-19 pneumonia cohort. Another 284 children with pneumonia caused by other viruses were defined as the viral pneumonia cohort. The epidemiologic, clinical, and laboratory findings were compared by Kolmogorov-Smirnov test, t-test, Mann-Whitney U test and Contingency table method. Drug usage, immunotherapy, blood transfusion, and need for oxygen support were collected as the treatment indexes. Mortality, intensive care needs and symptomatic duration were collected as the outcome indicators.

**Results:**

Compared with the viral pneumonia cohort, children in the COVID-19 cohort were mostly exposed to family members confirmed to have COVID-19 (53/64 vs. 23/284), were of older median age (6.3 vs. 3.2 years), and had a higher proportion of ground-glass opacity (GGO) on computed tomography (18/40 vs. 0/38, *P* < 0.001). Children in the COVID-19 pneumonia cohort had a lower proportion of severe cases (1/40 vs. 38/284, *P* = 0.048), and lower cases with high fever (3/40 vs. 167/284, *P* < 0.001), requiring intensive care (1/40 vs. 32/284, *P* < 0.047) and with shorter symptomatic duration (median 5 vs. 8 d, *P* < 0.001). The proportion of cases with evaluated inflammatory indicators, biochemical indicators related to organ or tissue damage, D-dimer and secondary bacterial infection were lower in the COVID-19 pneumonia cohort than those in the viral pneumonia cohort (*P* < 0.05). No statistical differences were found in the duration of positive PCR results from pharyngeal swabs in 25 children with COVID-19 who received antiviral drugs (lopinavir-ritonavir, ribavirin, and arbidol) as compared with duration in 39 children without antiviral therapy [median 10 vs. 9 d, *P* = 0.885].

**Conclusion:**

The symptoms and severity of COVID-19 pneumonia in children were no more severe than those in children with other viral pneumonia. Lopinavir-ritonavir, ribavirin and arbidol do not shorten the duration of positive PCR results from pharyngeal swabs in children with COVID-19. During the COVID-19 outbreak, attention also must be given to children with infection by other pathogens infection.

## Background

Coronaviruses are non-segmented positive-stranded RNA viruses, with protrusions on the surface resembling a corona, and with a roughly 30 kb genome surrounded by a protein envelope [1]. Three coronavirus disease outbreaks have happened in the past two decades: severe acute respiratory syndrome (SARS) [2] in 2003, Middle East respiratory syndrome (MERS) [3] in 2012 and now the Coronavirus Disease 2019 (COVID-19) [4]. Since the first case of COVID-19 was reported in Wuhan, China, in December 2019 until January 18, 2021, SARS-CoV-2 has infected more than 93 million individuals worldwide [5].

Generally, people from all age groups are susceptible to SARS-CoV-2. However, according to data published by the Chinese Center for Disease Control and Prevention (CCDC), the proportion of SARS-CoV-2 infection in children appears to be lower than that of adults [6]. Several studies have described the epidemiologic characteristics and clinical features of children with COVID-19, and showed that most children cases had a clear exposure history and the symptoms were milder than adult infection cases [7–9].

Until now, few studies have focused on the treatment of children with COVID-19 and how to identify serious cases during treatment. The similarities and differences between COVID-19 and other sources of viral pneumonia in children, in terms of disease onset, clinical manifestations, test characteristics, and treatment responses, remain to be elucidated. Therefore, we conducted a retrospective multi-center cohort study of acute viral pneumonia in children during the COVID-19 outbreak with the purpose of answering the following three questions: What are the clinical and epidemiological features of children with COVID-19? What do laboratory tests and imaging findings tell us about children with COVID-19 pneumonia as compared with viral pneumonia caused by other viruses? And, what are the differences in treatment experience and outcomes of COVID-19 as compared with those of other viral pneumonia in children?

## Methods

### Study design and participants

This is a retrospective multi-center cohort study. The study protocol was reviewed and approved by the Medical Ethics Committee of General Hospital of Southern Theater Command of Chinese People’s Liberation Army (approval number 2020–04). Signed informed consent of participants or their guardians was waived. Children with COVID-19 and pneumonia caused by other viruses diagnosed from December 15, 2019 to March 15, 2020 during COVID-19 outbreak from 20 hospitals in China were enrolled.

Diagnosis of COVID-19 pneumonia was based on guidelines issued by the National Health Commission of China [10]. Cases with positive results on quantitative RT-PCR tests for SARS-CoV-2 sampled by nasopharyngeal or throat swabs were enrolled into the COVID-19 cohort, of which 40 cases that developed pneumonia were defined as the COVID-19 pneumonia cohort.

The children with pneumonia caused by other viruses were enrolled into the viral pneumonia cohort. The inclusion criteria for the viral pneumonia cohort were: 1) Diagnosed with community acquired pneumonia (CAP) based on the guidelines for diagnosis and treatment of CAP in children (2019 version) [11]; 2) Laboratory virus testing confirmed existence of viruses associated with pneumonia, including virus or viral antigen in upper-respiratory specimens (e.g., nasopharyngeal aspirates) and lower respiratory samples (e.g., induced sputum) by culture, immunofluorescence microscopy or molecular diagnostic assays (such as PCR, RT-PCR), and on measurement of antibodies in serum samples; 3) Clinically-defined pneumonia mainly caused by the detected virus. The exclusion criteria were: 1) Patients older than 18 years old or younger than 28 days; 2) The initial pathogens of pneumonia were mycoplasma pneumonia (MP), bacterial pneumonia or others, and the detected viruses were considered as secondary infection.

### Data collection

Clinical data on epidemiology, signs and symptoms, underlying diseases, laboratory findings, disease diagnosis, and treatment records were retrospectively reviewed by physicians of the 20 hospitals. Data were recorded in a customized data collection form and were checked by two study investigators (RGL and XGQ) independently. Two experienced radiologists (at least 5 years of experience in chest CT) of each hospital were invited to review the X-ray and CT images, and make the case descriptions and radiological diagnoses using uniform standards.

Baseline patient data include age, gender, weight, underlying chronic disease and residential address. Clinical manifestations include respiratory symptoms and signs, gastrointestinal symptoms (diarrhea, vomiting, abdominal pain, and bloating), neurological symptoms (consciousness, response to stimulations, and convulsions), and systemic symptoms (fever, fatigue, and muscle aches). Laboratory findings include whole blood cell tests, biochemical laboratory tests (liver enzymes, creatine kinase (CK), creatine kinase-MB (CK-MB), and lactate dehydrogenase (LDH)), coagulation function (prothrombin time (PT), activated partial thromboplastin time (APTT), fibrinogen, and D-dimer), humoral immune function (IgG, IgA, and IgM), cellular immune function (CD4^+^/CD8^+^ T cells) and infection indicators (C-reactive protein (CRP), procalcitonin (PCT), erythrocyte sedimentation rate (ESR), interleukin-6 (IL-6)). PCT test value greater than 0.25 ng/ml was considered as elevated level [12]. The real-time reverse transcriptase polymerase chain-reaction (RT-PCR) test of SARS-CoV-2 was done as described previously [13] and was performed using nasopharyngeal swab, nasal swab and/or rectal swab at the local CCDC.

The severity of SARS-CoV-2 infections were classified as asymptomatic infection, mild infection (without pneumonia), moderate infection (with mild pneumonia), severe infection and critical infection, according to the diagnosis and treatment guidelines for COVID-19 (draft version 7) [10] issued by the National Health Commission of China. Also, the same criteria were used to assess the severity of other viral pneumonia (mild and severe pneumonia) in children.

Drug usage (antiviral drugs, interferon alpha, antibiotics, glucocorticoids, etc.), immunotherapy (immunoglobulin, immunomodulator, etc.), blood transfusion, and need for oxygen support were collected as the treatment indexes. Mortality, intensive care needs and symptomatic duration were collected as the outcome indicators.

### Statistical analysis

All statistical analysis was done using IBM SPSS (version 20, IBM Corp, Armonk, NY, USA). The Kolmogorov-Smirnov test was used to evaluate distribution type. Normally distributed data are expressed as mean ± standard deviation (mean ± SD) and independent sample *t*-test was used for comparisons between two cohorts. Non-normally distributed data and ordinal data are expressed as median (inter-quartile range) and Mann-Whitney *U* test was used to compare the differences between cohorts. Contingency table method was used to check the proportion of counts data. *P*-values less than 0.05 were considered as statistically significant.

## Results

**Fig. 1:**
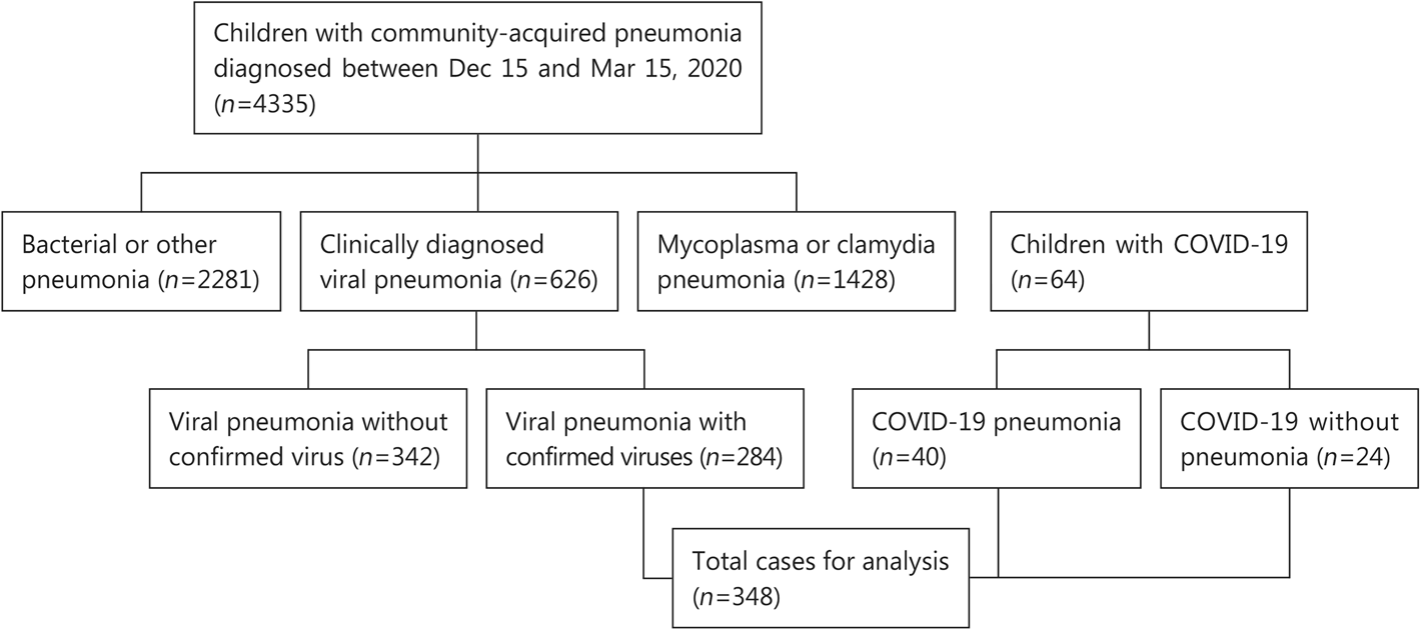
Flow diagram of recruiting participants. Total of 4335 cases of children CAP had been diagnosed in 20 hospitals, of which 626 cases were clinical diagnosed with viral pneumonia, and finally 284 cases with confirmed other viruses were included, and 64 cases COVID-19 children in the cohort

A total of 64 children patients were included in the COVID-19 cohort, among which 33 patients were from Shenzhen, six patients were from Guangzhou, ten patients were from Wuhan, ten patients were from Huangshi, four were from Zunyi and one was from Hangzhou. From December 15 to March 15, 2020, a total of 4335 cases of children with CAP have been diagnosed in the 20 hospitals, of which 626 cases were clinically diagnosed with viral pneumonia. Finally, 284 viral pneumonia cases with confirmed virus infection were included in the viral pneumonia cohort. The flow chart of participants’ recruitment is shown in Fig. 1.

The epidemiological and baseline characteristics of all 348 participants are shown in Table 1. Thirty-one children (48.4%) in the COVID-19 cohort and 161 cases (56.7%) in the viral pneumonia cohort were males. No differences were found between two cohorts, while the ages of children in the COVID-19 cohort were older (median 6.3 vs 3.2 years) than those in the viral pneumonia cohort. Two of 64 children in the COVID-19 cohort and 19 of 284 children in the viral pneumonia cohort had underlying chronic diseases. No significant differences were found in baseline characteristics between the two cohorts (*P* = 0.279). Contact with confirmed COVID-infected family members (53/64, 82.8%) was the main exposure mode of COVID-19 children. Household exposure was higher in the COVID-19 cohort than in the viral pneumonia cohort (53/64 vs. 23/284, *P* < 0.001). The prevalence of pneumonia in children with COVID-19 was 62.5% (40/64). The ratios of cases progressing from SARS-CoV-2 infection to pneumonia were 4/6 for children younger than 1 year old, 11/23 for 1–5 years old, 15/20 for 6–10 years old, and 10/15 for 11–18 years old. No significant differences were found in the ratios between the different age groups (*P* = 0.701).

**Table 1:** Epidemiological and baseline characteristics of over all 348 participants

General data	COVID-19 cohort (*n =* 64)	Viral pneumonia cohort (*n =* 284)	*P*
Age			
Median (range)	6.3 yr (3 mon to 18 yr)	3.2 yr (1 mon to 13 yr)	< 0.001
< 1 yr [*n* (%)]	6 (9.4)	44 (15.5)	< 0.001
1–5 yr [*n* (%)]	23 (35.9)	220 (77.5)	
6–10 yr [*n* (%)]	20 (31.2)	19 (6.7)	
11–18 yr [*n* (%)]	15 (18.8)	1 (0.4)	
Male [*n*(%)]	31 (48.4)	161 (56.7)	0.230
Underlying chronic disease [*n* (%)]	2 (3.1)	19 (6.7)	0.279
Chronic lung disease (repeated wheezing)	1 (1.6)	14 (4.9)	0.231
Heart disease	0 (0.0)	1 (0.4)	1.000
Others	1 (1.6)	5 (1.8)	1.000
Exposure history [*n* (%)]^a^			
Contacted with confirmed family members	53 (82.8)	23 (8.1)	< 0.001
Contacted with other confirmed cases	3 (4.7)	15 (5.3)	
Travel or residence in epidemic area^a^	7 (10.9)	0 (0.0)	
No clear exposure history	1 (1.6)	246 (86.6)	

^a^No epidemic area was defined for other respiratory viruses

To ensure comparability between cohorts, COVID-19 cases with pneumonia were filtered out (COVID-19 pneumonia cohort) to compare with the viral pneumonia cohort in terms of clinical manifestations, spectrum of disease severity, and laboratory and imaging findings (Table 2). No significant differences were found in gastrointestinal symptoms (1/40 vs. 17/284) and dry cough (6/40 vs. 35/284) between the two cohorts, but the proportions of cases with fever (37.3 °C, 22/40 vs. 221/284), high fever (> 39.0 °C, 3/40 vs. 167/284), cough with sputum production (0/40 vs. 229/284), rhinitis (1/40 vs. 130/284), and wheezing (0/40 vs. 43/284) were lower in the COVID-19 pneumonia cohort than in the viral pneumonia cohort (*P* < 0.05). In addition, the duration of fever was shorter in the COVID-19 pneumonia cohort than that in the viral pneumonia cohort [median (IQR), 2 (1–3.25) d vs. 4 (2–5) d, *P* = 0.004)].

**Table 2:** Symptoms, signs, disease severity, laboratory tests and radiographic findings of COVID-19 pneumonia and other viral pneumonia

Measures	COVID-19 pneumonia (*n =* 40)^a^	Viral pneumonia (*n =* 284)^a^	*P*
Symptoms and signs [*n* (%)]			
Fever	22 (55.0)	221 (77.8)	< 0.001
Duration of fever [d, median (IQR)]	2.00 (1.00–3.25)	4.00 (2.00–5.00)	0.004
Highest temperature [*n* (%)]			
< 37.3 °C	18 (45.0)	63 (22.2)	< 0.001
37.4–37.9 °C	8 (20.0)	8 (2.8)	
38.0–38.9 °C	11 (27.5)	46 (16.2)	
> 39.0 °C	3 (7.5)	167 (58.8)	
Cough [*n* (%)]	6 (15.0)	256 (90.1)	< 0.001
Dry cough [*n* (%)]	6 (15.0)	35 (12.3)	0.634
Cough with Sputum production [*n* (%)]	0 (0.0)	229 (80.6)	< 0.001
Sore throat [*n* (%)]	1 (2.5)	8 (2.8)	0.909
Rhinitis [*n* (%)]	1 (2.5)	130 (45.8)	< 0.001
Short of breath [*n* (%)]	1 (2.5)	22 (7.7)	0.226
Wheezing [*n* (%)]	0 (0.0)	43 (15.1)	0.017
Gastrointestinal symptoms [*n* (%)]	1 (2.5)	17 (6.0)	0.709
Fatigue or muscle aches [*n* (%)]	1 (2.5)	12 (4.2)	0.603
Neurological symptoms [*n* (%)]	2 (5.0)	15 (5.3)	0.940
Three depression sign [*n* (%)]	1 (2.5)	36 (12.7)	0.058
Low SaO_2_ of inhospital (< 95%,*n* (%)]	1 (2.5)	9 (3.2)	0.819
Severity [*n* (%)]			
Moderate	39 (97.5)	246 (86.6)	0.048
Server or critical	1 (2.5)	38 (13.4)	
Blood routine			
WBC count [×10^9^/L; median (IQR)]	5.3 (4.4–7.8)	8.6 (6.3–12.1)	< 0.001
< 5.5 × 10^9^/L [*n* (%)]	19 (47.5)	38 (13.4)	< 0.001
Neutrophil count [× 10^9^/L; median (IQR)]	2.5 (1.9–3.3)	3.2 (2.1–4.6)	0.029
< 1.1 × 109/L [*n* (%)]	3 (7.5)	12 (4.2)	0.356
Platelet count [×10^9^/L; median (IQR)]	253.0 (217.3–320.0)	275.0 (209.0–350.0)	0.410
< 120 × 10^9^/L [*n* (%)]	1 (2.5)	9 (3.2)	0.819
Inflammatory indicators [*n* (%)]			
PCT (> 0.25 ng/ml)	2/34 (5.9)	78/209 (37.3)	< 0.001
CRP (> 10 mg/L)	5/38 (13.2)	80/281 (28.5)	0.045
ESR (> 20 s)	5/36 (13.9)	35/62 (56.5)	< 0.001
IL-6 (> 20.9 ng/L)	5/34 (14.7)	62/168 (36.9)	0.012
Blood biochemistry			
LDH [U/L; median (IQR)]	210.0 (187.0–482.4)	349.0 (228.5–418.5)	< 0.001
> 300 U/L [*n* (%)]	13/26 (36.1)	131/265 (49.4)	< 0.001
ALT [U/L; median (IQR)]	12.5 (9.25–24.0)	17 (13–23)	0.035
> 45 U/L [*n* (%)]	4 (10.0)	18/275 (6.5)	0.432
AST [U/L; median (IQR)]	33.9 (19.8–41.3)	37.0 (31.0–45.0)	0.011
> 50 U/L [*n* (%)]	4 (10.0)	36/276 (13.0)	0.588
CK [U/L; median (IQR)]	70.0 (57.0–91.8)	110.5 (79.3–155.8)	< 0.001
> 185 U/L [*n* (%)]	2/35 (5.7)	48/276 (17.4)	0.076
CK-MB (> 27 U/L) [*n* (%)]	5/35 (14.3)	165/270 (61.1)	< 0.001
Humoral immunity [g/L; median (IQR)]			
Ig G	8.1 (4.8–10.6)	8.4 (6.9–10.2)	0.712
Ig M	1.3 (1.0–1.6)	1.3 (0.9–1.6)	0.995
Ig A	1.0 (0.5–1.3)	0.9 (0.6–1.4)	0.218
Cellular immunity			
CD4^+^/CD8^+^ T cell [median (IQR)]	1.2 (0.9–1.6)	1.2 (0.9–1.8)	0.534
< 0.96 [*n* (%)]	6/17 (35.3)	4/16 (25.0)	0.479
0.96–2.05 [*n* (%)]	10/17 (58.8)	9/16 (56.2)	
> 2.05 [*n* (%)]	1/17 (5.9)	3/16 (18.8)	
Coagulation			
Fibrinogen [g/L; median (IQR)]	2.5 (2.4–3.0)	3.1 (2.4–3.9)	0.114
D-dimer [mg/L; median (IQR)]	0.32 (0.25–0.42)	0.53 (0.33–1.00)	0.004
> 0.5 mg/L [*n* (%)]	7/35 (20.0)	18/29 (62.1)	0.001
Prothrombin time [s, median (IQR)]	11.2 (10.2–13.3)	11.2 (10.3–13.0)	0.113
APTT [s, median (IQR)]	34.7 (31.3–36.4)	31.6 (25.8–35.7)	0.111
Co-infection [*n* (%)]			
Virus	3 (7.5)	22 (7.7)	0.956
MP	9 (22.5)	61 (21.5)	0.883
Secondary-infection with bacteria [*n* (%)]	0 (0.0)	52 (18.3)	0.003
Affected area on radiography [*n* (%)]— no. (%)			
Left lung lobe	5 (12.5)	18/202 (8.9)	0.071
Right lung lobe	14 (35.0)	41/202 (20.3)	
Bilateral lung lobe	21 (52.5)	143/202 (70.8)	
CT images of the chest [*n* (%)]			
GGO	18 (45.0)	0/38 (0.0)	< 0.001
Tiny nodules	6 (15.0)	2/38 (5.3)	0.297
Consolidation	5 (12.5)	21/38 (55.3)	< 0.001
Consolidation combined with GGO	4 (10.0)	1/38 (2.6)	0.387
Cable shadow	11 (27.5)	18/38 (47.4)	0.070
Light shadow	6 (15.0)	5/38 (13.2)	0.815
Streak shadow	6 (15.0)	6/38 (15.8)	0.923
Hydrothorax	1 (2.5)	1/38 (2.6)	1.000

The denominator of the proportional count in the table was the total number of evaluated cases, and the numerator is the number of cases positive on this index

*WBC* White blood cell, *PCT* Procalcitonin, *CRP* C-reactive protein, *ESR* Erythrocyte sedimentation rate, *IL-6* Interleukin-6, *LDH* Lactate dehydrogenase, *ALT* Alanine aminotransferase, *AST* Aspartate aminotransferase, *CK* Creatine kinase, *CK-MB* Creatine kinase – MB, *APTT* Activated partial thromboplastin time, *MP* Mycoplasma pneumonia, *GGO* Ground-glass opacity

^a^ Missing data are present by a fraction

Only one child was in critical condition in the COVID-19 pneumonia cohort, with clinical manifestations including shortness of breath, neurological symptoms (drowsiness), three signs of depression, low blood oxygen saturation and increased PCT, CRP, D-dimer and CK-MB on admission, and had received intubation and invasive ventilator-assisted ventilation immediately after admission.

The proportion of children who developed severe conditions was lower in the COVID-19 pneumonia cohort than in the viral pneumonia cohort (1/40 vs. 38/284, *P* = 0.048). However, no significant differences were found in pneumonia subgroups caused by respiratory syncytial virus (RSV) (1/40 vs. 19/133, *P* = 0.041) and human adenovirus (1/40 vs. 7/25, *P* = 0.002) as compared to those with pneumonia caused by influenza A and B (1/40 vs. 5/57, *P* = 0.182) or parainfluenza virus (1/40 vs. 3/29, *P* = 0.168). The comparison between children with COVID-19 pneumonia and pneumonia caused by the other four common types of respiratory viruses is shown in Table 3.

**Table 3:** The comparison between pneumonia caused by SARS-COV-2 and other four common kinds of respiratory viruses^*^

Measures	COVID-19 pneumonia (*n =* 40)	RSV pneumonia (*n =* 133)	Influenza A and B pneumonia (*n =* 57)	Parainfluenza virus pneumonia (*n =* 29)	Human adenovirus pneumonia (*n =* 25)
Age					
Median (yr)	7.0	2.6	4.0	3.4	3.9
< 1 yr [*n* (%)]	4 (10.0)	25 (18.8)	6 (10.5)	5 (17.2)	1 (4.0)
1–5 yr [*n* (%)]	11 (27.5)	108 (81.2)	42 (73.7)	23 (79.3)	17 (68.0)
6–10 yr [*n* (%)]	15 (37.5)	0 (0.0)	8 (14.0)	1 (3.4)	7 (28.0)
11–18 yr [*n* (%)]	10 (25.0)	0 (0.0)	1 (1.8)	0 (0.0)	0 (0.0)
Male [*n* (%)]	18 (45.0)	73 (54.9)	30 (52.6)	19 (65.5)	16 (64.0)
Underlying chronic disease [*n* (%)]	2 (5.0)	10 (7.5)	3 (5.3)	1 (3.4)	2 (8.0)
Chronic lung disease (repeated wheezing)	1 (2.5)	5 (3.8)	2 (3.5)	1 (3.4)	2 (8.0)
Heart disease	0 (0.0)	1 (0.8)	0 (0.0)	0 (0.0)	0 (0.0)
Others	1 (2.5)	4 (3.0)	1 (1.8)	0 (0.0)	0 (0.0)
Symptoms and signs					
Fever [*n* (%)]	22 (55.0)	105 (78.9)	49 (86.0)	15 (51.7)	22 (88.0)
Duration of fever [d, median (IQR)]	2 (1–3)	3 (2–5)	4 (2.5–6.0)	3 (2–4)	5 (2.00–8.25)
Highest temperature [*n* (%)]					
< 37.3 °C	18 (45.0)	28 (21.0)	8 (14.0)	14 (48.3)	3 (12.0)
37.4–37.9 °C	8 (20.0)	7 (5.3)	0 (0.0)	0 (0.0)	0 (0.0)
38.0–38.9 °C	11 (27.5)	23 (17.3)	12 (21.0)	4 (13.8)	0 (0.0)
> 39.0 °C	3 (7.5)	75 (56.4)	37 (64.9)	11 (37.9)	22 (88.0)
Cough [*n* (%)]	6 (15.0)	119 (89.5)	53 (93.0)	26 (89.6)	19 (76.0)
Dry cough [*n* (%)]	6 (15.0)	12 (9.0)	8 (14.0)	1 (3.4)	6 (24.0)
Cough with sputum production [*n* (%)]	0 (0.0)	119 (89.5)	45 (78.9)	25 (86.2)	13 (52.0)
Sore throat [*n* (%)]	1 (2.5)	2 (1.5)	5 (8.8)	0 (0.0)	1 (4.0)
Rhinitis [*n* (%)]	1 (2.5)	65 (48.9)	21 (36.8)	18 (62.1)	8 (32.0)
Short of breath [*n* (%)]	1 (2.5)	13 (9.8)	3 (5.3)	1 (3.4)	2 (8.0)
Wheezing [*n* (%)]	0 (0.0)	28 (21.0)	3 (5.3)	2 (6.9)	2 (8.0)
Gastrointestinal symptoms [*n* (%)]	1 (2.5)	7 (5.3)	4 (7.0)	2 (6.9)	1 (4.0)
Fatigue or muscle aches [*n* (%)]	1 (2.5)	2 (1.5)	10 (17.5)	0 (0.0)	0 (0.0)
Neurological symptoms [*n* (%)]	2 (5.0)	8 (6.0)	6 (10.5)	0 (0.0)	0 (0.0)
Three depressions sign [*n* (%)]	1 (2.5)	20 (15.0)	3 (5.3)	3 (10.3)	5 (20.0)
Low SaO_2_ of inhospital (≤95%) [*n* (%)]	1 (2.5)	4 (3.0)	1 (1.8)	1 (3.4)	3 (12.0)
Symptom duration [D; median (IQR)]	5 (0–8)	8 (5–10)	8 (5–10)	9 (6–11)	9 (4–12)
Severity [*n* (%)]					
Moderate	39 (97.5)	114 (85.7)	52 (91.2)	26 (89.7)	18 (72.0)
Server or critical	1 (2.5)	19 (14.3)	5 (8.8)	3 (10.3)	7 (28.0)
Blood routine					
WBC [×10^9^/L; median (IQR)]	5.3 (4.4–7.8)	8.6 (6.3–11.5)	7.0 (5.7–9.8)	7.7 (6.0–12.3)	9.3 (6.9–14.3)
< 5.5 × 10^9^/L [*n* (%)]	19 (47.5)	17 (12.8)	14 (24.6)	4 (13.8)	2 (8.0)
Neutrophil count [×10^9^/L,median (IQR)]	2.5 (1.9–3.3)	3.5 (2.2–4.8)	2.3 (1.6–3.9)	3.3 (2.6–4.9)	3.0 (1.8–4.4)
< 1.1 × 10^9^/L [*n* (%)]	3 (7.5)	0 (0.0)	10 (17.5)	0 (0.0)	1 (4.0)
Platelet count [× 10^9^/L, median (IQR)]	253.0 (217.2.0–320.0)	285.0 (224.0–366.8)	246.5 (187.3–298.5)	262.0 (173.5–325.0)	259.0 (193.0–313.8)
< 120 × 10^9^/L [*n* (%)]	1 (2.5)	5 (3.8)	2 (3.5)	1 (3.4)	1 (4.0)
Infection biomarkers [*n* (%)]					
PCT (> 0.25 ng/ml)	2/34 (5.9)	28/88 (31.8)	15/46 (32.6)	7/19 (36.8)	14/21 (66.7)
CRP (> 10 mg/L)	5/38 (13.2)	25/131 (19.1)	24 (42.1)	4/28 (14.3)	16 (64.0)
ESR (> 20 s)	5/36 (13.9)	12/22 (54.5)	10/18 (55.6)	1/5 (20.0)	9/12 (7.5)
IL-6 (> 20.9 ng/L)	5/34 (14.7)	25/80 (31.2)	9/31 (29.0)	6/17 (35.3)	15/23 (65.2)
Blood biochemistry					
LDH [U/L, median (IQR)]	210.0 (187.0–482.4)	361.0 (303.5–418.5)	363.5 (269.5–489.8)	310.5 (288.0–361.8)	342.0 (286.0–405.0)
> 300 U/L [*n* (%)]	13/36 (36.1)	97/125 (77.6)	33/50 (66.0)	18/28 (64.3)	17 (68.0)
ALT [U/L, median (IQR)]	12.5 (9.25–24.0)	18.0 (14.0–24.0)	15.0 (12.3–20.8)	16.5 (13.0–19.0)	15.5 (9.3–19.3)
> 45 U/L [*n* (%)]	4 (10.0)	7/130 (5.4)	3/56 (5.4)	0/28 (0.0)	5/24 (20.8)
AST [U/L, median (IQR)]	33.9 (19.8–41.3)	38.5 (33.0–46.0)	39.0 (28.5–46.8)	36.5 (32.3–42.3)	29.0 (23.0–37.5)
> 50 U/L [*n* (%)]	4 (10.0)	19/130 (14.6)	8/56 (14.3)	2/28 (7.1)	1 (4.0)
CK [U/L, median (IQR)]	70.0 (57.0–91.8)	113.0 (82.0–154.3)	122.5 (88.0–179.0)	110.5 (84.3–181.0)	86.0 (49.0–154.5)
> 185 U/L [*n* (%)]	2/35 (5.7)	23/130 (17.7)	12/56 (21.4)	7/28 (25.0)	4 (16.0)
CK-MB (> 27 U/L) [*n* (%)]	5/35 (14.3)	87/126 (69.0)	21/56 (37.5)	22 (75.9)	15/24 (62.5)
Humoral immunity [g/L, median (IQR)]					
Ig G	8.1 (4.8–10.6)	8.4 (6.4–10.2)	8.4 (6.9–10.6)	8.5 (6.5–10.6)	8.9 (7.2–9.9)
Ig M	1.3 (1.0–1.6)	1.3 (0.8–1.8)	1.2 (1.1–1.5)	1.3 (1.0–1.8)	1.3 (0.9–1.5)
Ig A	1.0 (0.5–1.3)	0.8 (0.5–0.1.3)	0.9 (0.5–1.4)	0.9 (0.6–1.4)	1.0 (0.7–1.2)
Co-infection [*n* (%)]					
Virus^*^	3 (7.5)				
Mycoplasma pneumoniae	9 (22.5)	21 (15.8)	16 (28.1)	2 (6.9)	9 (36.0)
Secondary-infection with Bacteria [*n* (%)]	0 (0.0)	25 (18.8)	6 (10.5)	6 (20.7)	5 (20.0)
Affected area on radiography [*n* (%)] — no. (%)					
Left lung lobe	5 (12.5)	2/81 (2.5)	9/52 (17.3)	2/23 (8.7)	0/17 (0.0)
Right lung lobe	14 (35.0)	16/81 (19.7)	17/52 (32.7)	3/23 (13.0)	1/17 (5.9)
Bilateral lung lobe	21 (52.5)	63/81 (77.8)	26/52 (50.0)	18/23 (78.3)	16/17 (94.1)
CT images of the chest [*n* (%)]					
GGO	18 (45.0)	0/12 (0.0)	0/9 (0.0)	0/2 (0.0)	0/7 (0.0)
Tiny nodules	6 (15.0)	0/12 (0.0)	1/9 (11.1)	0/2 (0.0)	0/7 (0.0)
Consolidation	5 (12.5)	2/12 (16.7)	8/9 (88.9)	1/2 (50.0)	4/7 (57.1)
Consolidation combined with GGO	4 (10.0)	1/12 (8.3)	0/9 (0.0)	0/2 (0.0)	0/7 (0.0)
Cable shadow	11 (27.5)	5/12 (41.7)	4/9 (44.4)	0/2 (0.0)	5/7 (71.4)
Light shadow	6 (15.0)	1/12 (8.3)	2/9 (22.2)	1/2 (50.0)	0/7 (0.0)
Streak shadow	6 (15.0)	5/12 (41.7)	0/9 (0.0)	0/2 (0.0)	0/7 (0.0)
Hydrothorax	1 (2.5)	0/12 (0.0)	0/9 (0.0)	0/2 (0.0)	1/7 (14.3)

*. Missing data are present by a fraction. WBC. White blood cell. PCT. Procalcitonin. CRP. C-reactive protein. ESR. Erythrocyte sedimentation rate. IL-6. Interleukin-6. LDH. Lactate dehydrogenase. ALT. Alanine aminotransferase. AST. Aspartate aminotransferase. CK. Creatine kinase. CK-MB. Creatine kinase – MB. RSV. respiratory syncytial virus. GGO. Ground-glass opacity. *The blank in the column of co-infection was because viral pneumonia caused by multiple viruses was not included in this comparison. Of the 284 cases of viral pneumonia, 133 were caused by RSV, 57 were caused by influenza A or influenza B, 29 were caused by parainfluenza virus, 25 were caused by human adenovirus, 22 were caused by multiple viruses, six were caused by human rhinovirus, six were caused by human Boca virus and six cases were caused by other viruses. The comparison was made between pneumonia caused by SARS-COV-2 and other four common kinds of respiratory viruses

On admission, counts of white blood cells and lymphocytes of COVID-19 pneumonia children were lower than those of other viral pneumonia (*P* < 0.05). The proportion of cases with leucopenia (white blood cell counts < 5.5 × 10^9^/L) was higher in the COVID-19 cohort (19/40 vs. 38/284). Levels of LDH, ALT, AST, CK, D-dimer, and the proportion of cases with elevated LDH (13/36 vs. 131/265), CK-MB (5/35 vs. 165/270), PCT (2/34 vs. 78/209), CRP (5/38 vs. 80/281), ESR (5/36 vs. 35/62), IL-6 (5/34 vs. 62/168) and D-dimer (> 0.5 mg/L, 7/35 vs. 18/29) were lower in the COVID-19 pneumonia cohort as compared with the viral pneumonia cohort (*P* < 0.05). No significant differences were noted between the two cohorts in co-infection with other viruses (3/40 vs. 22/284, *P* = 0.956) or co-infection with MP (9/40 vs. 61/284, *P* = 0.883). However, secondary-infection with bacteria was less frequently detected in the COVID-19 pneumonia cohort (0/40 vs. 52/284, *P* = 0.003).

Ground-glass opacity (GGO) was the most common radiographic presentation of children with COVID-19 pneumonia. The proportion of cases with GGO was significantly higher (18/40 vs. 0/38, *P* < 0.001), while the proportion of cases with consolidation (5/40 vs. 21/38, *P* < 0.001) was lower in the COVID-19 pneumonia cohort as compared to the viral pneumonia cohort. Other radiographic presentations of COVID-19 pneumonia included tiny nodules, consolidation combined with GGO, cable shadow, light shadow, streak shadow and hydrothorax, for which no significant differences were found between COVID-19 pneumonia cohort and the viral pneumonia cohort (Table 2).

Treatments and outcomes of children with COVID-19 pneumonia and other viral pneumonia are listed in Table 4. The proportion of children who received lopinavir–ritonavir, ribavirin and interferon alfa was higher in the COVID-19 pneumonia cohort, but the proportion of children who received oseltamivir was lower, as compared with that in the viral pneumonia cohort (*P* < 0.05). The proportion of children with COVID-19 pneumonia who received antibiotics (8/40 vs. 261/284, *P* < 0.001), corticosteroids (1/40 vs. 50/284, *P* = 0.014), immunoglobulin (1/40 vs. 39/284, *P* = 0.043) and who needed oxygen support (*P* = 0.024) were lower than that of those with other viral pneumonia.

**Table 4:** Treatments and outcome of COVID-19 pneumonia and other viral pneumonia

Measures	COVID-19 pneumonia (*n =* 40)	Viral pneumonia (*n =* 284)	*P*
Antiviral therapy [Yes, *n* (%)]	14 (35.0)	71 (25.0)	0.178
Lopinavir–ritonavir	9 (22.5)	0 (0.0)	< 0.001
Ribavirin	4 (10.0)	2 (0.7)	< 0.001
Arbidol	1 (2.5)	0 (0.0)	0.252
Oseltamivir	1 (2.5)	65 (22.9)	0.003
Paramive	0 (0.0)	4 (1.4)	1.000
Others	0 (0.0)	3 (1.1)	1.000
Interferon alfa [*n* (%)]	38 (95.0)	15 (5.3)	< 0.001
Antibiotic therapy [Yes, *n* (%)]	8 (20.0)	261 (91.9)	< 0.001
None	32 (80.0)	23 (8.1)	< 0.001
One kind	4 (10.0)	151 (53.2)	
Two kinds	3 (4.7)	99 (34.9)	
Three or more kinds	1 (2.5)	11 (3.9)	
Corticosteroids [*n* (%)]	1 (2.5)	50 (17.6)	0.014
Immunoglobulin [*n* (%)]	1 (2.5)	39 (13.7)	0.043
Blood transfusion [*n* (%)]	1 (2.5)	4 (1.4)	1.000
Oxygen support [*n* (%)]			
None	39 (97.5)	238 (83.8)	0.024
Nasal catheter, mask or other	0 (0.0)	22 (7.7)	
Non-invasive ventilation	0 (0.0)	8 (2.8)	
Invasive mechanical ventilation	1 (2.5)	16 (5.6)	
Mortality [*n* (%)]	0 (0.0)	1 (0.4)	1.000
Required ICU support [*n* (%)]	1 (2.5)	32 (11.3)	0.047
Symptomatic duration [d, median (IQR)]	5 (0–8)	8 (4–12)	< 0.001
Hospital stay [d, median (IQR)]^a^	15 (10–23)	6 (5–8)	< 0.001

^a^The duration of hospital stay was not used as a prognostic indicator in this study, because many children with COVID-19 were observed in the hospital after the symptoms completely disappeared for the possibility of infectivity

**Fig. 2:**
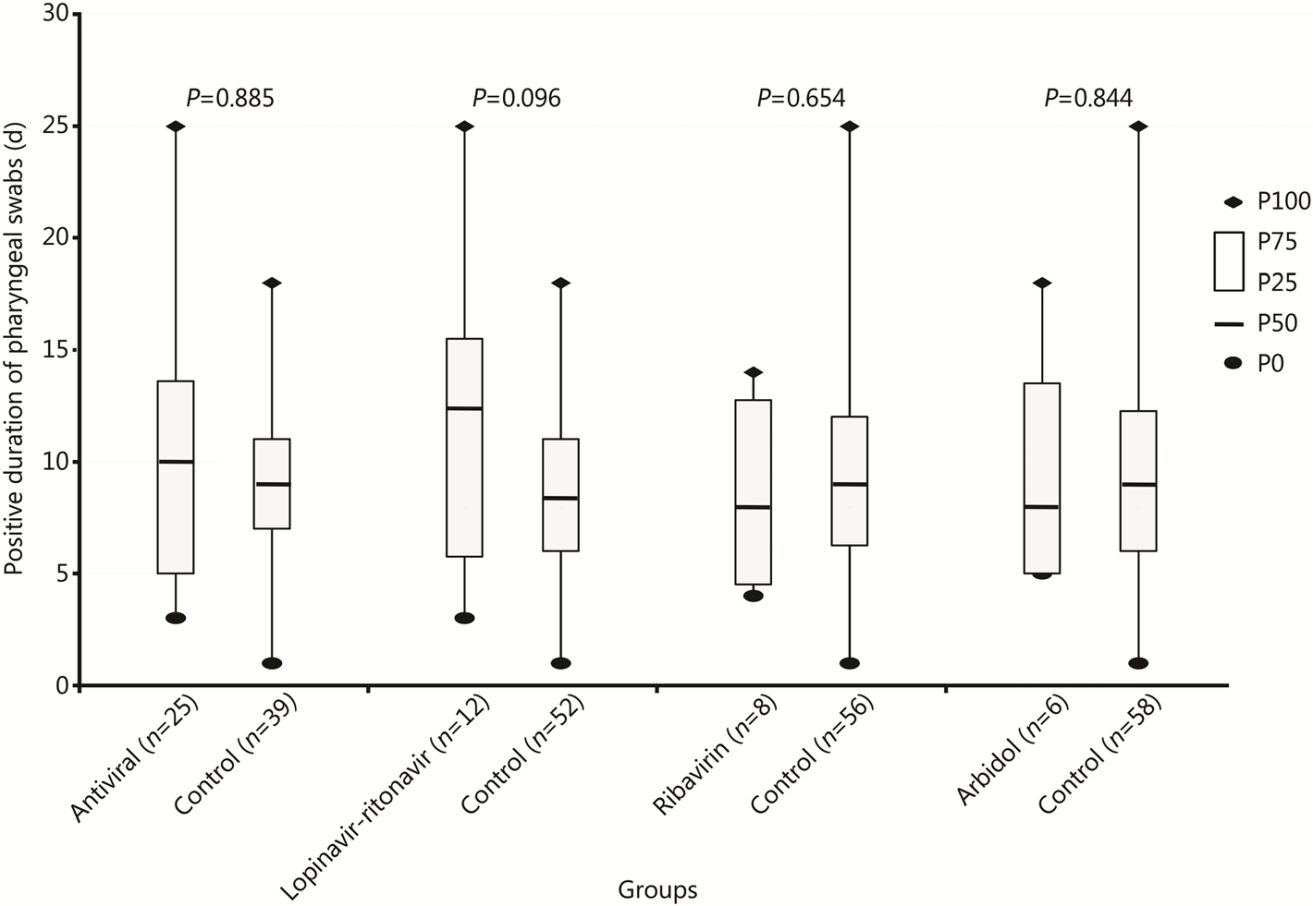
Effects of lopinavir–ritonavir, ribavirin and arbidol on children with COVID-19. Lopinavir-ritonavir, ribavirin, and arbidol were used in about 1/3 of children with COVID-19, but they did not shorten the duration of positive PCR results from pharyngeal swabs for children with COVID-19

Evaluation of the effects of three antiviral drugs through comparisons within the COVID-19 cohort showed that lopinavir–ritonavir (lopinavir 6–10 mg/kg and ritonavir 1.5–2.5 mg/kg per day, median for 9 days) was used in 12 (18.8%) cases, ribavirin (10–15 mg/kg per day, median for 8 days) was used in 8 (12.5%) cases, and arbidol (5–8 mg/kg per day, median for 7 days) was used in 6 (9.4%) children with COVID-19. The duration of positive PCR results from pharyngeal swabs was not significantly different between 25 COVID-19 children receiving those antiviral drugs and that of 39 control cases not receiving those antiviral drugs [median (IQR), 10 (5–13.5) d vs 9 (7–11) d, *P* = 0.885]. Subgroup analysis performed to evaluate the effectiveness of certain antiviral drugs showed that no significant differences were found in the duration of positive PCR results from pharyngeal swabs in children with COVID-19 who used lopinavir–ritonavir, ribavirin, or arbidol with non-antiviral therapy controls (*P* < 0.05, Fig. 2).

Up until April 15, 2020, only one patient in the viral pneumonia cohort died and the other 347 patients were discharged, with no significant differences in the mortality rate between the two cohorts. As compared with the viral pneumonia cohorts, the proportion of cases that required intensive care was lower (1/40 vs. 32/284, *P* = 0.047) and the symptomatic duration was shorter [median (IQR), 5 (0–8) d vs. 8 (4–12) d, *P* < 0.001] in the COVID-19 pneumonia cohort.

## Discussion

Results of this multi-center retrospective cohort study provided new insight into the clinical features and treatment of children with COVID-19. Comparison of the data of 64 children with COVID-19 with the data of 284 children patients with other types of viral pneumonia identified differences in clinical manifestations, laboratory parameters, imaging characteristics and treatment. COVID-19 children had a lower proportion of severe cases, lower cases with high fever, cases requiring intensive care and overall shorter symptomatic duration. Cases with inflammatory indicators, biochemical indicators related to organ or tissue damage, D-dimer and secondary bacterial infection were also lower in the COVID-19 pneumonia. However, no significant differences were found between groups in the duration of positive PCR results from pharyngeal swabs after receiving antiviral treatment.

Respiratory illnesses are consistently troublesome for children in the winter months and early spring. SARS-CoV-2 is an emerging contagious pathogen causing a high prevalence of pneumonia. Understanding the clinical manifestations in children with COVID-19, especially those different from other similar diseases, is important for diagnosis, treatment and management of this disease. Clear exposure histories, GGO on CT images are the main characteristics of SARS-CoV-2 infection in children with COVID-19, and most children (82.8%) in the present study cohort were from family cluster cases; however, family clusters of pneumonia were not common among other virus infections, because most adults already have immunity against other common respiratory viruses and will not develop pneumonia. GGO on CT images was a distinct characteristic of children with COVID-19, and did not occur in the other 284 viral pneumonia cases. However, the proportion of the occurrence of GGO was obviously lower than that of adult cases [14], and 55% of COVID-19 pneumonia cases were without these typical radiographic changes. Moreover, it was observed in the present study that, after several days, GGO can transform into lesions that cannot differentiate COVID-19 from other lung infections, which makes it more difficult to identify children with COVID-19 by using CT images. Consolidation was more common in the CT images of other viral pneumonia cases, but this cannot be used to exclude COVID-19, as consolidation can be found in 12.5% of pediatric COVID-19 pneumonia cases. The epidemiology and the PCR tests are therefore essential in the diagnosis of COVID-19.

The prevalence of pneumonia in SARS-CoV-2 infection was 62.5%, similar with that of SARS (65%) [15], but much higher than that of H1N1 influenza (11%) and many other viruses. No differences were reported in the proportion of children in different age groups who developed pneumonia after SARS-CoV-2 infection, and this situation can be observed in the research of Dong et al. [16]. and Wu et al. [6], indicating that younger age has no protective effect on preventing SARA-CoV-2 infection from developing COVID-19 pneumonia in children. Children in the present study were usually well cared for at home and had relatively fewer opportunities to be exposed to people with serious transmissible infection, which may help to explain the lower incidence of children with COVID-19 compared with adults.

The symptoms and disease severity of COVID-19 pneumonia in children were no more severe than other common viral pneumonia. Fever and cough were the most common symptoms of children with COVID-19 pneumonia. In the present study, the proportion of cases with fever among children with COVID-19 was significantly lower than that of adults with COVID-19 [13, 17], and that of children with other viral pneumonia. Fever temperatures were mainly low to moderate, and the proportion of cases with high fever was only 7.5%, lower than that of other viral pneumonia cases (58.8%); the duration of fever was also shorter in children with COVID-19 than those in other viral pneumonia. The proportion of severe cases (1/40) in children with COVID-19 pneumonia in the present study is consistent with previous results reported by Lu et al. [9]. (3 of 171 children with COVID-19) and Dong et al. [16]. (21 of 731 children with COVID-19), which were significantly lower than those of adult cases [18] and lower than pneumonia caused by RSV (19/133) and human adenovirus (5/25). Also, the proportion of cases that required intensive care was lower and the symptomatic duration was short as compared with the viral pneumonia cohort. Therefore, we concluded that COVID-19 pneumonia in children was no more severe than pneumonia caused by common viruses from community exposure.

Uncontrolled inflammatory innate responses may lead to tissue and organ damage in SARS-CoV-2 infection [19]. High levels of cytokines were detected in adult patients with severe illness caused by SARS-CoV [20], MERS-CoV [21], and SARS-CoV-2 [18] infections. In addition, excessively increased inflammatory indicators (CRP, PCT) were observed in severe pneumonia cases caused by human adenovirus [22]. The only single case of critical COVID-19 among children in our study, had significantly elevated IL-6 (120.31 ng/L), IL-10 (33.38 ng/L), and PCT (0.43 ng/ml), and acute respiratory distress syndrome, acute renal failure and cardiac insufficiency occurred. However, compared to pneumonia caused by other viruses, we found that the inflammatory indicators (CRP, PCT, ESR, IL-6, LDH), as well as biochemical indicators associated with organ or tissue damage (ALT, AST, CK, CK-MB), and indexes associated with disseminated intravascular coagulation, were lower in the children with COVID-19 pneumonia. This indicates that SARS-CoV-2 infection has less effect on excessive activation of the innate immune system in children and rarely triggered cytokine storm, which may be associated with milder clinical manifestations of children with COVID-19. The only single severe case in the COVID-19 pneumonia cohort was combined with hydronephrosis caused by kidney stones (1/1 vs. 1/39, *P* = 0.037), and five severe cases in the other viral pneumonia cohort were found to have underlying chronic disease (5/38 vs. 15/246, *P* = 0.112). However, we cannot make any conclusion on the effects of comorbidities on progression to severe pneumonia.

Antiviral therapy, corticosteroids, immune therapy and antibiotics usage were the main components of COVID-19 treatment. By activating innate immunity, interferon was nebulized in more than 90% of children in the COVID-19 cohort in the present study as well as in those with other viral infections [23]. However, this study failed to evaluate the effects of interferon on SARS-CoV-2 infection. Lopinavir-ritonavir, ribavirin, and arbidol were used in about 1/3 of children with COVID-19 in total, but they did not shorten the duration of positive PCR results from pharyngeal swabs for children with COVID-19. For the other viral pneumonia, oseltamivir and paramivir were used in influenza A and influenza B related pneumonia, but antiviral drugs were rarely used in pneumonia caused by other viruses. Since all 64 children with COVID-19 had a relatively good prognosis, we proposed that it was not necessary to aggressively administer antiviral drugs in children with COVID-19 before effective and safe antiviral drugs are confirmed. Nevertheless, several studies have shown that immune therapy may be beneficial in dealing with cytokine storm syndrome in adults with severe COVID-19 [24]. As mentioned above, cytokine storms were relatively rare and inflammatory indicators were lower in children with COVID-19 as compared to other viral pneumonia, so corticosteroids were not recommended as for other community-acquired viral pneumonia. However, corticosteroids might be tried for critical children with COVID-19 with obvious cytokine reactions, acute respiratory distress syndrome, multiple organ damage or with obvious wheezing. The percentage of viral-viral co-infection was 7.5% and viral-MP co-infection was 22.5% for children with COVID-19 pneumonia, which were similar between the viral pneumonia cohort and that reported by other studies on childhood viral pneumonia [25]. Secondary-infection with bacteria was detected in 18.5% cases of the viral pneumonia cohort, whereas none was found in the COVID-19 pneumonia cohort. Few cases were given penicillin for prophylactic treatment of possible bacterial infection. Milder symptoms and shorter symptomatic duration in children with COVID-19 may explain the low rate of secondary bacterial infections. For children with COVID-19, antibiotics are not used routinely, but multiple pathogen tests are recommended because of the potential of high proportions of co-infection in children with viral pneumonia.

The major limitation of our study was the selection bias. First, not all children with viral pneumonia were included since clinicians failed to identify causative agents in some individuals with pneumonia. Although all participating hospitals routinely tested pathogens in children with pneumonia, the relatively severe cases had more chances of pathogens being detected. Secondly, although the 64 SARS-CoV-2 infected patients contained cases from both Hubei province and outside Hubei province, their representativeness was limited due to a limited sample size.

## Conclusions

Younger age has no protective effect on preventing SARS-CoV-2 infection from developing into COVID-19 pneumonia in children. The symptoms and severity of children with COVID-19 pneumonia were no more severe than that of other viral pneumonia. Lopinavir-ritonavir, ribavirin, and arbidol do not shorten the duration of positive PCR results from pharyngeal swabs in children with COVID-19. Multi-respiratory-pathogen tests, including SARS-COV-2, are necessary and even during a COVID-19 outbreak, children with infection from other pathogens require sufficient attention.

## Data Availability

The datasets used and/or analyzed during the present study are available from the corresponding author on reasonable request.

## Abbreviations

APTT: Activated partial thromboplastin time
CAP: Community acquired pneumonia
CCDC: Chinese Center for Disease Control and Prevention
CK: Creatine kinase
CK-MB: Creatine kinase-MB
COVID-19: Coronavirus disease-2019
CRP: C-reactive protein
CT: Computed tomography
ESR: Erythrocyte sedimentation rate
GGO: Ground-glass opacity
IL-6: Interleukin-6
LDH: Lactate dehydrogenase
MERS: Middle East respiratory syndrome
MP: Mycoplasma pneumonia
PCR: Polymerase chain-reaction
PCT: Procalcitonin
PT: Prothrombin time
RSV: Respiratory syncytial virus
SARS: Severe acute respiratory syndrome
WBC: White blood cell
